# Atrial Fibrillation Recurrence After Catheter Ablation Is Associated with TAG72 Protein

**DOI:** 10.3390/jcdd13010039

**Published:** 2026-01-09

**Authors:** Karapet V. Davtyan, Aishat A. Abdullaeva, Nadezhda G. Gumanova, Natalya L. Bogdanova, Hacob A. Brutyan, Elena N. Kalemberg, Ekaterina V. Bazaeva, Maria S. Kharlap, Svetlana E. Serdyuk, Oksana M. Drapkina

**Affiliations:** National Medical Research Center for Therapy and Preventive Medicine, Ministry of Health of Russia, 101000 Moscow, Russia; kdavtyan@gnicpm.ru (K.V.D.); nlbogdanova@gnicpm.ru (N.L.B.); abrutyan@gnicpm.ru (H.A.B.); etsareva@gnicpm.ru (E.N.K.); ebazaeva@gnicpm.ru (E.V.B.); mkharlap@gnicpm.ru (M.S.K.); sserdyuk@gnicpm.ru (S.E.S.); odrapkina@gnicpm.ru (O.M.D.)

**Keywords:** atrial fibrillation, catheter ablation, pulmonary veins isolation, biomarker, TAG72, atrial fibrillation recurrence

## Abstract

Despite the efficacy of catheter ablation in preventing recurrences of atrial fibrillation (AF), the reasons for its lack of success in some patients remain unknown. The aim of this study was to try to identify a new predictor of AF recurrence following catheter-based treatment. This prospective study enrolled a cohort of patients with AF. Based on the results of a one-year follow-up, patients were divided into two groups: Group 1 (“vein-dependent” AF)—patients who achieved a successful outcome after 1–2 catheter ablation procedures—and Group 2 (“non-vein-dependent” AF)—patients with confirmed complete pulmonary vein isolation (PVI) or with an identified “non-vein-dependent” AF substrate. Blood samples were collected prior to the procedure and biobanked. Initial proteomic profiling of the serum using protein microarrays identified several candidate proteins, whose elevated levels were subsequently confirmed by an enzyme-linked immunosorbent assay (ELISA). This article presents data on one such protein—TAG72. A comparison of TAG72 levels (%OD normalized units) between Group 1 (“vein-dependent” AF) and Group 2 (“non-vein-dependent” AF) revealed a statistically significant increase in the latter group (128.9 [98.2; 284.4] vs. 84.3 [73.8; 92.1], *p* < 0.001). These data provide the first evidence implicating TAG72 in the pathogenesis of AF.

## 1. Introduction

Atrial fibrillation (AF) is the most common cardiac arrhythmia worldwide, imposing a significant burden on patient health [1]. It is associated with various risk factors, including structural heart disease, metabolic disorders, and lifestyle factors such as obesity and alcohol consumption. AF carries a substantial socioeconomic burden, leading to increased morbidity, mortality, and healthcare costs due to frequent hospitalizations and the need for continuous monitoring (Abdullaeva et al., 2024; Aldaas et al., 2019) [2,3]. The economic impact is further compounded by the necessity for long-term therapy and frequent medical interventions related to AF (Zakynthinos et al., 2024) [4].

Catheter ablation has emerged as a cornerstone treatment for AF, offering improved quality of life and clinical outcomes. However, its efficacy can vary, underscoring the need to continue the search for predictors of a successful procedural outcome (R. Providencia et al., 2024) [5]. The inability to guarantee success for all patients highlights the necessity for a personalized approach to selecting candidates for interventional treatment. The ongoing search for reliable predictors of efficacy is crucial for optimizing patient outcomes.

Numerous studies are actively investigating laboratory markers as potential predictors of successful catheter ablation in AF. In recent years, researchers have explored the association between AF recurrence post-ablation and proteins such as RAD51 and p63. Elevated serum levels of RAD51 and p63 were found in patients with “non-vein-dependent” (substrate-related) AF (Gumanova et al., 2024) [6]. Prior to this, an association with only oncological diseases had been described for the RAD51 and p63 proteins [7,8]. The literature contains several articles providing a detailed description of the protocol for analyzing labeled serum proteins using antibody microarrays [9,10].

Fibrosis biomarkers may also be useful for predicting successful AF ablation by helping to identify patients with extensive fibrotic remodeling, who may have less favorable outcomes (An et al., 2017) [11]. Furthermore, studies have established that combining biomarkers such as NT-proBNP, hs-CRP, and IL-6 with clinical parameters (left atrial size and patient history) may help improve the accuracy of predicting AF recurrence (Mo et al., 2024; Kimura et al., 2014) [12,13].

TAG72 (Tumor-Associated Glycoprotein 72) is a glycoprotein primarily described in the literature as a target for the diagnosis and treatment of oncological diseases [14,15,16,17].

TAG72 is a high molecular weight, tumor-associated glycoprotein (mucin-like) defined by the monoclonal antibody B72.3. Its molecular structure and association with various pathological processes make it a subject of active investigation. The expression level of TAG72 is linked to tumor aggressiveness and its ability to modulate immune responses. Emerging research also suggests its potential involvement in cardiovascular diseases, opening prospects for its use as a biomarker in cardiology. Thus, the clinical utility of TAG72 currently extends beyond oncology.

The aim of the present study was to identify a biomarker associated with AF recurrence following a catheter ablation procedure, based on one year of patient follow-up.

## 2. Materials and Methods

### 2.1. Patient Selection Criteria and Study Design

A single-center prospective study was conducted at the National Medical Research Center for Therapy and Preventive Medicine between 2017 and 2019, enrolling 198 patients. The follow-up period lasted until 2021. This study was registered in the ClinicalTrials.gov registry (Identifier: NCT05170607). The study was conducted in accordance with the Declaration of Helsinki and was approved by the Center’s Independent Ethics Committee (Approval Code—01-06/17, Approval Date—2 February 2017).

All participants provided written informed consent prior to enrollment.

**Figure 1:**
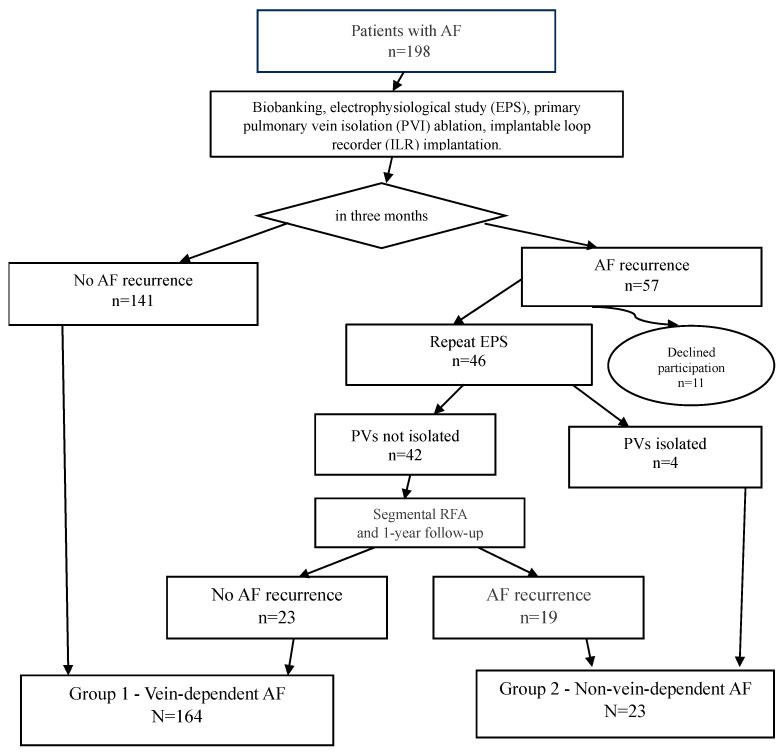
Study design.

The study design is as follows: All 198 patients from the cohort (the study flowchart is presented in Figure 1) underwent cryoballoon pulmonary vein isolation with simultaneous implantation of a subcutaneous cardiac rhythm monitor. The follow-up protocol included scheduled examinations at 3, 6, and 12 months post-procedure.

#### 2.1.1. Patient Cohorts and Follow-Up Protocol

During the first three months of follow-up, atrial fibrillation recurrence was recorded in 57 patients. A total of 11 patients were excluded from further analysis due to protocol violations: refusal to participate in blood biobanking, failure to attend scheduled follow-up examinations, refusal to undergo repeat interventions, or relocation outside the study region.

To verify the efficacy of pulmonary vein isolation (PVI), an electrophysiological study (EPS) was performed on 46 patients. In 4 patients, durable PVI was confirmed, allowing their classification into the substrate-dependent AF group. The remaining 42 patients underwent a repeat procedure—segmental radiofrequency ablation targeting the areas of conduction recovery (isolation of the pulmonary veins ostia only, without any additional interventions in the left atrium and right atrium). It was the second stage of treatment.

All patients included in the study (both after the initial and repeat procedures) continued follow-up according to the standard protocol with visits at 3, 6, and 12 months. Based on the results of the one-year follow-up, patients were distributed into two groups according to the mechanism of their arrhythmia.

The “vein-dependent” AF cohort (*n* = 164) comprised patients that had no AF recurrence. This group included 141 patients with a successful outcome after a single cryoablation procedure and 23 patients who experienced no further AF recurrences after a repeat procedure (segmental RF ablation).

The “non-vein-dependent” (substrate-dependent) AF cohort (*n* = 23) included patients with AF recurrences despite achieved pulmonary vein isolation. This cohort consisted of 4 patients with confirmed durable PVI and recurrences documented during the follow-up EPS, and 19 patients with persistent AF after two ablation procedures. The criterion for inclusion in this group was the confirmation of a pulmonary vein isolation block or the identification of an alternative, non-venous arrhythmogenic substrate.

#### 2.1.2. Inclusion and Exclusion Criteria

The study enrolled patients aged >18 years with paroxysmal or persistent AF and symptoms scoring 2b–4 on the EHRA scale, corresponding to standard indications for PVI. Exclusion criteria encompassed decompensated comorbidities (hypertension, diabetes mellitus, unstable coronary artery disease, chronic heart failure), active phase of systemic diseases, intracardiac thrombosis, left atrial (LA) size > 50 mm, left ventricular ejection fraction (LVEF) < 40%, mitral regurgitation ≥ grade 3, and interventricular septal thickness > 14 mm. Prior to inclusion, each patient provided written informed consent to participate in the study.

### 2.2. Research Methods

In accordance with the approved protocol, all study participants underwent a standardized preoperative examination. The laboratory diagnostic panel was aimed at identifying potential contraindications to the procedure and included complete blood count, comprehensive metabolic panel, urinalysis, and thyroid-stimulating hormone level assessment. A suite of instrumental investigations was performed for a comprehensive assessment of cardiopulmonary status and the condition of the upper gastrointestinal tract. This included the following: 12-lead resting ECG, chest X-ray in two projections, esophagogastroduodenoscopy, transthoracic echocardiography, and 24 h Holter ECG monitoring.

#### 2.2.1. Blood Sample Collection and Processing

Blood for laboratory analysis was collected from all patients from a peripheral vein in the preoperative period (either on the day of the intervention or one day prior) after an overnight fast. The obtained samples were centrifuged at 1000× *g* for 15 min at 4 °C to separate the serum. Aliquots of serum and plasma were stored at −27 °C until analysis. Prior to proteomic profiling, the serum samples were centrifuged again at 10,000× *g* for 15 min at 4 °C to pellet any potential cryoprecipitates. The protein concentration in the samples was measured spectrophotometrically using a NanoDrop One instrument (Thermo Scientific, Waltham, MA, USA) by assessing the optical density at 260 nm and 280 nm wavelengths, with human serum albumin used as the calibration standard.

#### 2.2.2. Analysis of Labeled Serum Proteins by Antibody Microarrays

Serum proteins were diluted to a concentration of 1 mg/mL in phosphate-buffered saline (PBS; Arrayit Corp., Sunnyvale, CA, USA), mixed with 20 µL of labeling buffer, and labeled by coupling to the Green 540 reagent (1 µL; Arrayit Corp.) for 60 min on ice. The coupling reaction was terminated by adding a stop solution (10 µL; Arrayit Corp.). Excess dye was removed via gel filtration. Labeled proteins (500 ng per slide in blocking buffer containing 3% dry milk) were processed using Explorer antibody microarrays (ASB600, Full Moon Biosystems, Sunnyvale, CA, USA). The arrays contained 656 antibodies per slide in duplicate. The complete antibody list is available at https://www.fullmoonbio.com/datasheets/ASB600_AbList.xls (accessed on 10 October 2025). Following a 1 h incubation and subsequent washing, the slides were incubated with a coupling buffer containing 3% dry milk for 2 h, washed again, and dried using a microcentrifuge for glass slides.

#### 2.2.3. Microarray Slide Scanning and Image Processing

Microarray slides were scanned and processed using an InnoScan 900 microarray scanner (Innopsys, Carbonne, France) to ensure positive signal correlation with corresponding controls on each slide. Image analysis was performed using Mapix software version 7.0.0 (Innopsys, Carbonne, France). Protein identification was based on spot position using microarray-specific Gal files cross-referenced with UniProtKB/Swiss-Prot database identifiers. The background signal at the reference wavelength was subtracted, and image intensity was expressed as the median pixel intensity. Spots exhibiting high-intensity variability between duplicates (coefficient of variation, CV > 25%) were excluded from subsequent analysis.

#### 2.2.4. Quantification of Serum TAG72 by ELISA

The concentration of TAG72 in serum samples was quantified using a commercial sandwich enzyme-linked immunosorbent assay (ELISA) kit (Catalog # MBS288813, MyBioSource, San Diego, CA, USA) according to the manufacturer’s protocol. The assay was performed within a concentration range of 0.312–20 ng/mL, with a sensitivity limit of 0.12 ng/mL. All samples were analyzed in duplicate. The assay precision was <8% for intra-plate and <12% for inter-plate coefficients of variation (CV).

Optical density (OD) was measured at 450 nm using 620 nm as the reference wavelength on a Tecan Infinite 200Pro plate reader (Switzerland). Since the measured concentrations fell below the kit’s standard calibration range, results are presented in normalized units (%) and calculated as follows: (OD450 of sample/mean OD450 of all samples) × 100.

Normalized Optical Density (%OD) is a standardized unit calculated as (OD of sample/mean OD of all samples) × 100. This method standardizes data and allows for relative concentration comparisons over time or between samples.

#### 2.2.5. Statistical Analysis

Statistical analysis and data visualization were performed using the R environment for statistical computing, version 4.5.1 (R Foundation for Statistical Computing, Vienna, Austria).

Descriptive statistics are presented as absolute and relative frequencies for categorical variables, and as mean and median (1st; 3rd quartiles) for quantitative variables. Normality of distribution for quantitative variables was assessed using the Anderson–Darling test, along with evaluation of the skewness coefficient (using an absolute value >1.96 as the critical threshold) and visual assessment using quantile–quantile (Q-Q) plots.

When estimating the prevalence of binary outcomes, exact binomial 95% confidence intervals (95% CI) were calculated.

For comparing groups regarding quantitative variables, the Brunner–Munzel test was used. The 95% CI for the median difference was estimated using nonparametric bootstrapping (B = 1000). For comparing groups regarding binary outcomes, Fisher’s exact test with mid-p adjustment was used. The odds ratio (OR) with its corresponding 95% CI was used as a measure of association strength for binary outcomes (the Haldane–Anscombe correction was applied in cases of zero cells in a 2 × 2 contingency table). For comparing groups regarding categorical variables with more than two levels, Pearson’s chi-square test was used. For comparative analysis of ordinal variables, univariate proportional odds models with likelihood ratio tests were employed.

To measure the strength of association between a binary outcome and potential quantitative predictors, odds ratios (OR) with corresponding 95% CIs were estimated using univariate logistic regression models (log_2_ transformation was applied to covariates with pronounced left-skewed distribution).

Differences between groups and associations were considered statistically significant at *p* < 0.05.

For quantitative predictors, ROC analysis was also performed. This included estimating the Area Under the Curve (AUC) and the optimal cutoff value using Youden’s J statistic, followed by an assessment of predictive accuracy, sensitivity, specificity, and positive and negative predictive values with corresponding 95% CIs.

## 3. Results

The study analyzed a cohort of 198 patients with diagnosed atrial fibrillation (age range: 19–80 years) who received treatment at the Cardiology Department of the National Medical Research Center for Therapy and Preventive medicine. The sample consisted of 63% (*n* = 114) men and 37% (*n* = 74) women. By the final follow-up point, data were available for 187 patients, of which 164 cases were classified as vein-dependent and 23 as non-vein-dependent.

### Clinical and Demographic Parameters of Patients

**Cohort description.** The study cohort (*n* = 198) comprised 114 men (57.6%) and 84 women (42.4%). Age parameters were as follows: median current age 59 years [51.00–65.00], mean age at arrhythmia onset 53.1 ± 10.6 years; median AF duration was 3.5 years. According to the EHRA scale, 172 patients (86.8%) had high (class 2b, *n* = 83) or very high (class 3, *n* = 89) arrhythmia-related symptom burden. The mean CHA_2_DS_2_-VASc thromboembolic risk score was 2. The mean left atrial (anteroposterior) size was 42.5 mm [39.0–45.0].

Among comorbid conditions, controlled arterial hypertension was the most prevalent (*n* = 149). Other significant conditions included stable coronary artery disease (*n* = 15, including post-infarction cardiosclerosis [PICS]—*n* = 3), a history of cerebrovascular events (stroke: *n* = 14; transient ischemic attack [TIA]: *n* = 8), and impaired glucose metabolism (controlled type 2 diabetes mellitus: *n* = 28; impaired glucose tolerance: *n* = 16). Systemic diseases were diagnosed in 12 patients (myasthenia gravis, psoriasis, gout, history of lymphogranulomatosis). Comprehensive clinical and demographic data are presented in Table 1.

Comparative analysis of the groups demonstrated no statistically significant differences in key demographic and clinical parameters. Specifically, the groups were comparable in terms of sex (*p* = 0.373), age (*p* = 0.858), and body mass index (BMI, *p* = 0.919). The prevalence of comorbid conditions, including diabetes mellitus, arterial hypertension, and obesity, also showed no significant differences (*p* = 1.0; *p* = 0.802; *p* = 0.595, respectively). Regarding echocardiographic parameters, no statistically significant differences were found between the groups: left atrial size (*p* = 0.092), left ventricular ejection fraction (*p* = 0.243), and left ventricular end diastolic dimension (LVEDD, *p* = 0.972).

Among laboratory parameters, the level of the heart failure marker NT-proBNP was significantly higher in the non-vein-dependent group compared to the vein-dependent group: 678.0 pg/mL versus 250.5 pg/mL, respectively (*p* = 0.005).

The TAG72 quantification results, presented in Table 2, are reported in one unit of measurement: the percentage of the solution’s optical density (%).

**Figure 2:**
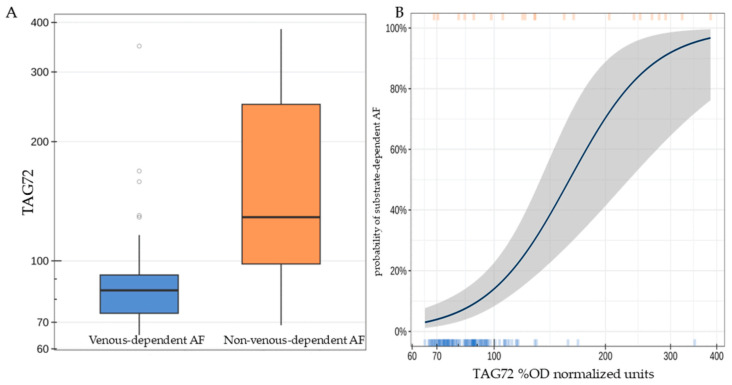
TAG72 level (%OD normalized units) in patients of the studied groups (**A**); probability of detecting substrate-dependent atrial fibrillation depending on TAG72 level. The highlighted area indicates the deviation range of TAG72 levels (**B**).

Patients with substrate-dependent AF were characterized by a statistically significantly higher concentration of TAG72 (the median difference was 44.6 [95% CI: 13.8; 126.3], *p* < 0.001, Figure 2A). A twofold increase in TAG72 level was associated with an average 14.5-fold increase [95% CI: 5.25; 54.4] in the odds of detecting substrate-dependent AF (*p* < 0.001, Figure 2B).

**Figure 3:**
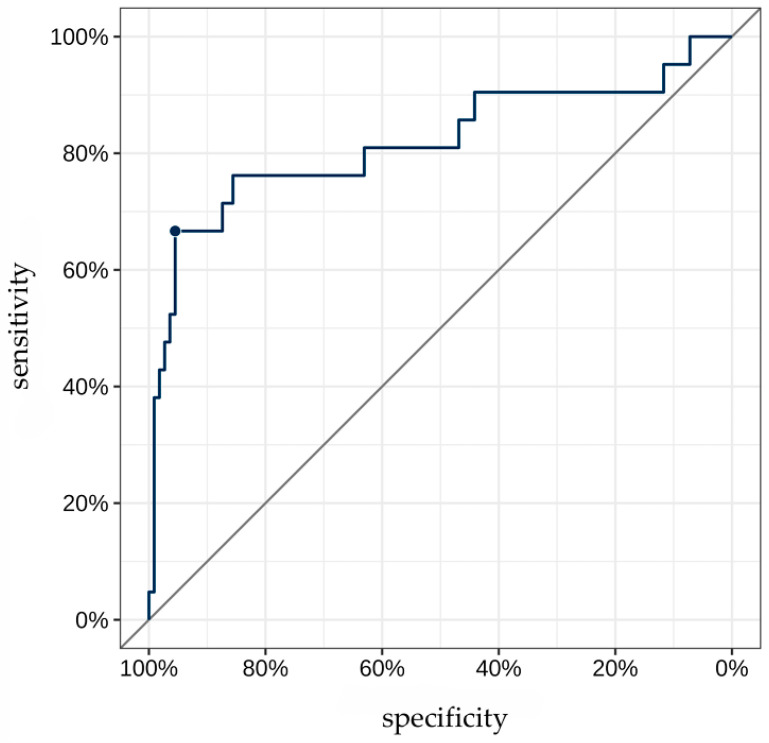
ROC curve for TAG72 level as a predictor of “non-venous-dependent” atrial fibrillation. The highlighted point on the ROC curve corresponds to the optimal diagnostic threshold of TAG72 level for detecting substrate-based atrial fibrillation. At this value, the best balance is achieved between high sensitivity and specificity.

**Figure 4:**
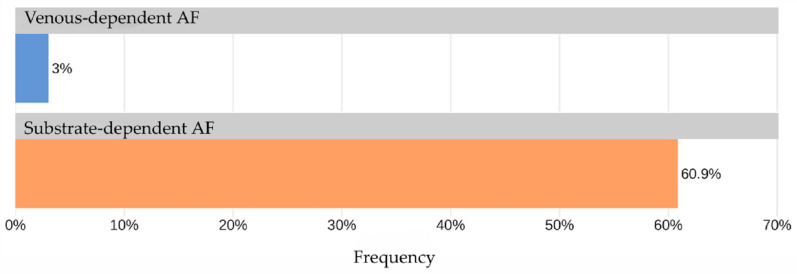
Frequency of TAG72 > 117.75 in the study groups.

The AUC for TAG72 concentration as a predictor for identifying “non-venous-dependent” AF was 0.82 [95% CI: 0.69; 0.95] (Figure 3). A TAG72 level above 117.75 (the optimal cutoff determined by Youden’s J statistic) was associated with 42.4-fold higher odds [95% CI: 11.8; 152] of detecting “non-venous-dependent” AF (*p* < 0.001, Figure 4).

The predictive accuracy of this cutoff was 90.9% [95% CI: 84.7; 95.2], with a sensitivity of 66.7% [95% CI: 43; 85.4] and a specificity of 95.5% [95% CI: 89.8; 98.5]. The positive predictive value (PPV) and negative predictive value (NPV) were 73.7% [95% CI: 48.8; 90.9] and 93.8% [95% CI: 87.7; 97.5], respectively.

The odds of detecting “non-venous-dependent” atrial fibrillation were significantly higher with elevated TAG72 levels across several thresholds.

Table 3 presents the results of a comparative analysis of the study groups regarding TAG72 levels.

The conducted analysis demonstrates that the concentration of the TAG72 is a powerful and statistically significant predictor of the presence of substrate-dependent atrial fibrillation (AF).

**Clinical Significance:**

TAG72 level is a highly informative biomarker for diagnosing “non-venous-dependent” AF. The >117.75 cutoff can be considered optimal for comprehensive diagnostics, providing the best balance between identifying true-positive cases and minimizing false negatives. Low TAG72 levels (%OD normalized units) (<90) can reliably rule out the presence of non-paroxysmal AF with high confidence, while high levels (>150) indicate its presence with maximum specificity.

## 4. Discussion

This finding has significant potential implications for stratifying patients with AF. Elevated TAG72 expression may serve as a prognostic marker, suggesting a high probability of a non-venous-dependent AF form. This could influence treatment strategy, as patients anticipated to have low efficacy from catheter ablation may require more extensive interventions or a focus on pharmacological therapy.

Put simply, the higher the TAG72 protein level, the lower the probability that a patient’s atrial fibrillation is triggered by pulmonary vein foci, and the greater the likelihood that its origin involves a different mechanism, such as an inflammatory process.

The principal finding of our study is an association between elevated levels of the glycoprotein TAG72 and a therapy-resistant form of atrial fibrillation. A novel finding of this work is the association between elevated TAG72 levels and a form of AF with a pathogenesis that appears independent of pulmonary vein triggers. Elevated TAG72 levels have been previously documented in the literature in association with various malignancies. This biomarker is primarily linked to gastric cancer, colorectal carcinoma, and gynecological neoplasms, where its serum levels serve as a potential tool for diagnosis and monitoring. For instance, tissue TAG72 expression in gastric cancer has been reported to range from 8.4 to 562.9 pmol/g, with higher concentrations observed in mucinous adenocarcinomas (Chung et al., 1995) [18]. Furthermore, pre-operative serum TAG72 levels demonstrate considerable variability, from 3 to 357 U/mL, exceeding a threshold of 6 U/mL in 46.8% of patients (Allende et al., 2000) [19].

In contrast to these oncological findings, our study cohort did not exhibit a similarly pronounced elevation in serum TAG72 levels that would be indicative of an underlying malignant process.

These results suggest that TAG72 could serve as a novel and potentially significant biomarker in patients with AF. Traditionally, pulmonary vein isolation via catheter ablation is a highly effective procedure for patients with a trigger-dependent form of the arrhythmia. However, the presence of an AF substrate unrelated to the pulmonary veins is a common cause of recurrence and procedural failure. The association we discovered positions the assessment of TAG72 expression as a potential tool for the pre-procedural identification of patients at high risk of recurrence due to the presence of such a substrate.

Consequently, the identification of such patients becomes a prerequisite for optimizing therapy. This is underscored by recent trial data. As highlighted by the randomized ERASE-AF trial, a strategy combining pulmonary vein isolation with individualized substrate modification targeting low-voltage myocardial areas (PVI + SM) demonstrated superior efficacy in preventing atrial arrhythmia recurrence in patients with persistent atrial fibrillation compared to PVI alone (hazard ratio 0.62; *p* = 0.006). This study underscores the critical importance of addressing the atrial substrate beyond the pulmonary veins for improving long-term outcomes in this challenging patient population [20]. Clinical trial data exists where a comprehensive pulsed field ablation strategy targeting both the pulmonary veins and atrial substrate achieved acute AF termination in 95.8% of patients and a 74.6% single-procedure success rate [21]. This finding has the potential to directly influence clinical practice and ablation strategy by enabling a shift toward a biomarker-guided, stratified approach. Elevated TAG72 levels could serve as a preoperative tool to identify patients with a high likelihood of substrate-dependent AF. For this specific subgroup, the cited evidence suggests that pulmonary vein isolation (PVI) alone may be insufficient.

Although the direct mechanisms of TAG72 involvement in arrhythmogenesis require further detailed investigation, several plausible explanations can be proposed. Given its known role as a mucin-like glycoprotein involved in cell adhesion and intercellular interactions, elevated TAG72 expression may promote structural atrial remodeling. This could manifest as activation of fibrosis, impairment of myocardial conductive properties, and the creation of a pro-arrhythmogenic substrate that sustains re-entry mechanisms. Furthermore, its suggested immunomodulatory function, noted in oncological research, may point to a role of local inflammation in the pathological process. Chronic inflammation is a well-established factor in the development and maintenance of AF, and TAG72 may act as a novel mediator of this process in the myocardium.

This study has several limitations. First, its observational design establishes an association but does not prove causation. Second, the sample size should be expanded to confirm these findings. To definitively establish the role of TAG72, further fundamental research is required to elucidate the precise molecular pathways through which this protein affects the electrophysiological properties of cardiomyocytes and the process of fibrogenesis. Additionally, further investigation into the association of TAG72 levels with comorbid inflammatory conditions, left atrial size, and the duration of atrial fibrillation prior to treatment initiation merits further investigation.

## Figures and Tables

**Table 1:** Clinical and demographic characteristics of the patients (*n* = 198).

Characteristics	Meaning
Demographic characteristics	
Men, *n* (%)	114 (57.6)
Age, Med [Q1–Q3]	59 [51.0–65.0]
Age of arrhythmia manifestation, years, M ± SD	53.1 ± 10.6
Body mass index, kg/m^2^, M ± SD	30.5 ± 4.63
AF parameters	
Duration of AF history, years Med [Q1–Q3]	3.5 [2.0–7.0]
Form of AF, paroxysmal, *n* (%)	151 (76.3)
Symptoms (EHRA)	
Class I, *n* (%)	8 (4.1%)
Class IIa, *n* (%)	17 (8.6%)
Class IIb, *n* (%)	83 (42.1%)
Class III, *n* (%)	89 (45.2%)
Risk scales	
Score for assessing the risk of stroke and systemic thromboembolism in patients with atrial fibrillation, points, Med [Q1–Q3]	2 [1–3]
Atrial fibrillation bleeding risk assessment scale	1 [0–2]
Echocardiographic parameters	
Anteroposterior dimension of the left atrium, mm, Med [Q1–Q3]	42.5 [39.0–45.0]
Left ventricular ejection fraction %, Med [Q1–Q3]	62 [59.0–66.0]
End diastolic dimension of the LV, mm, Med [Q1–Q3]	51 [48.0–54.5]
Comorbid conditions	
Arterial hypertension, *n* (%)	149 (75.6%)
Ischemic heart disease, *n* (%)	15 (7.6%)
Including history of myocardial infarction, *n* (%)	3 (1.5%)
History of acute cerebrovascular accident, *n* (%)	14 (7.1%)
History of transient ischemic attack (TIA), *n* (%)	8 (4.1%)
Type 2 diabetes mellitus, *n* (%)	28 (14.2%)
Impaired glucose tolerance (IGT), *n* (%)	16 (8.1%)
Systemic diseases, *n* (%)	12 (6.1%)

Data are presented as M ± SD (mean ± standard deviation), Med [Q1–Q3] (median [1st quartile–3rd quartile]), and *n* (%) (number of patients, percentage). LV—left ventricle, LA—left atrium. EHRA—European Heart Rhythm Association classification of AF symptoms.

**Table 2:** Comparative characteristics of the study groups.

Characteristics	Vein-Dependent AF (*n* = 164)	Non-Vein-Dependent AF (*n* = 23)	*p*
Sex:			
Women	68 (41.5%)	12 (52.2%)	0.373
Men	96 (58.5%)	11 (47.8%)	
Age (years), Med [IQR]	59 [52; 65]	57 [51; 65]	0.858
Body mass index, kg/m^2^, M (SD)	30.3 (4.61)	30.2 (4.15)	0.919
Echocardiographic parameters			
Anteroposterior dimension of the left atrium, mm, Med [IQR]	42 [39; 45]	43 [41.5; 47]	0.092
LV EF %, Med [IQR]	62 [59.8; 66]	61 [58.5; 64]	0.243
End diastolic dimension of the LV, mm, Med [IQR]	52 [48; 55]	51 [48.5; 54.5]	0.972
Comorbidities			
Type 2 diabetes mellitus, *n* (%)	22 (13,4%)	3 (13%)	1.0
Arterial hypertension, *n* (%)	122 (74.40%)	18 (78.3%)	0.802
Obesity	83 (50.6%)	13 (56.5%)	0.595
Laboratory parameters			
TAG72%OD Normalized units, Med [IQR]	84.3 [73.8; 92.1]	128.9 [98.2; 248.4]	<0.001
NT-proBNP, pg/mL, Med [IQR]	250.5 [76.4; 573]	678.0 [205; 1055.5]	0.005 *
hs-CRP, mg/L, Med [IQR]	2.15 [1.06; 5.68]	2.74 [1.20; 5.12]	0.864

* Differences were considered statistically significant at *p* < 0.05.

**Table 3:** The level of TAG72 in patients of the study groups.

Characteristics	“Venous-Dependent AF”	“Non-Venous Dependent AF”	*p*
TAG72	84.3 (73.8; 92.1)	128.9 (98.2; 248.4)	<0.001
TAG72 > 88	41 (36.9%)	17 (81%)	<0.001
TAG72 > 90	32 (28.8%)	16 (76.2%)	<0.001
TAG72 > 100	16 (14.4%)	15 (71.4%)	<0.001
TAG72 > 119	5 (4.5%)	14 (66.7%)	<0.001
TAG72 > 117.75	5 (4.5%)	14 (66.7%)	<0.001
TAG72 > 150	3 (2.7%)	10 (47.6%)	<0.001

## Data Availability

The original contributions presented in this study are included in the article.

## Abbreviations

The following abbreviations are used in this manuscript:

AF	Atrial fibrillation
ELISA	Enzyme-linked immunosorbent assay
EPS	Electrophysiological study
LA	Left atrium
LVEF	Left ventricular ejection fraction
OD	Optical density
OR	Odds ratio
PVI	Pulmonary vein isolation
PICS	Post-infarction cardiosclerosis
TAG72	Tumor-Associated Glycoprotein 72
TIA	Transient ischemic attack
